# Knock-out of a mitochondrial sirtuin protects neurons from degeneration in *Caenorhabditis elegans*

**DOI:** 10.1371/journal.pgen.1006965

**Published:** 2017-08-18

**Authors:** Rachele Sangaletti, Massimo D’Amico, Jeff Grant, David Della-Morte, Laura Bianchi

**Affiliations:** 1 Department of Physiology and Biophysics, University of Miami, Miller School of Medicine, Miami, Florida, United States of America; 2 Department of Systems Medicine, University of Rome Tor Vergata, Rome, Italy; 3 Department of Neurology, University of Miami, Miller School of Medicine, Miami, Florida, United States of America; 4 San Raffaele Roma Open University, Rome, Italy; University of California San Francisco, UNITED STATES

## Abstract

Sirtuins are NAD⁺-dependent deacetylases, lipoamidases, and ADP-ribosyltransferases that link cellular metabolism to multiple intracellular pathways that influence processes as diverse as cell survival, longevity, and cancer growth. Sirtuins influence the extent of neuronal death in stroke. However, different sirtuins appear to have opposite roles in neuronal protection. In *Caenorhabditis elegans*, we found that knock-out of mitochondrial sirtuin *sir-2*.*3*, homologous to mammalian SIRT4, is protective in both chemical ischemia and hyperactive channel induced necrosis. Furthermore, the protective effect of *sir-2*.*3* knock-out is enhanced by block of glycolysis and eliminated by a null mutation in *daf-16*/FOXO transcription factor, supporting the involvement of the insulin/IGF pathway. However, data in *Caenorhabditis elegans* cell culture suggest that the effects of *sir-2*.*3* knock-out act downstream of the DAF-2/IGF-1 receptor. Analysis of ROS in *sir-2*.*3* knock-out reveals that ROS become elevated in this mutant under ischemic conditions in dietary deprivation (DD), but to a lesser extent than in wild type, suggesting more robust activation of a ROS scavenging system in this mutant in the absence of food. This work suggests a deleterious role of SIRT4 during ischemic processes in mammals that must be further investigated and reveals a novel pathway that can be targeted for the design of therapies aimed at protecting neurons from death in ischemic conditions.

## Author summary

Sirtuins are NAD^+^-dependent enzymes that play key roles in neuronal death. However, the contribution of sirtuins to this cellular process is controversial with results supporting that activation or inhibition are protective depending on the cellular context and type of sirtuin. In this manuscript we report that knock-out of *Caenorhabditis elegans* mitochondrial sirtuin *2*.*3*, homolog of mammalian SIRT4, spares neurons from ischemic neuronal death and that this protective effect is enhanced by block of glycolysis. Furthermore, we report that ROS is eliminated more efficiently in *sir-2*.*3* knock-out as compared to wild type, under dietary deprivation. We conclude that inhibition of mammalian SIRT4 combined with dietary deprivation must be explored as a novel therapeutic approach to prevent ischemic damage.

## Introduction

Sirtuins function as NAD^+^-dependent deacetylases, lipoamidases, and or ADP-ribosyltransferases that control the acetylation, lipoylation, and ADP-ribosylation levels of several substrates in the cell, effectively linking nutrient availability to cellular processes [1,2]. Sirtuin-mediated modification of substrates results in the regulation of many different intracellular pathways that control various processes such as degeneration, ageing, and cancer growth [3–8]. The most studied of the 7 mammalian sirtuins is SIRT1. SIRT1 is activated by resveratrol [9], the anti-oxidant contained in red wine, and its activation has been linked to protection against ischemia [6,10–13], Huntington disease [14], Aß toxicity [15] and ageing across species [16]. For example, knock-out of *sir-2*.*1* abolishes life-extension mediated by caloric restriction [17]. Though this effect seems dependent on the conditions [18–22]. However, contradicting results support that activation or inhibition of sirtuins may be protective depending on the cellular context and type of sirtuin. For example, inhibition of SIRT2 protects against degeneration induced by α-synuclein toxicity [23] and treatment with sirtuin inhibitor sirtinol protects cultured cortical neurons against cell death in a model of excitotoxicity [24]. Moreover, it was shown that resveratrol is neuroprotective or neurotoxic depending on the concentrations used [11,24].

*Caenorhabditis elegans* has been instrumental for deciphering the molecular underpinning of cell death by necrosis and apoptosis [25,26]. In *Caenorhabditis elegans* neuronal death can be induced in the 6 touch sensing neurons by a genetic mutation that induces hyperactivation of the Na^+^/Ca^2+^ channel MEC-4 of the DEG/ENaC family (*mec-4(d)*) [27–29]. *mec-4(d)*-induced degeneration displays salient morphological and molecular features of neuronal death by necrosis that follows ischemia, including swelling of the cell body and processes, hyperactivation of a DEG/ENaC channel, elevation of intracellular Ca^2+^ concentration, and activation calpains and cathepsins [28–33]. Importantly, studies in mammals confirm a conserved mechanism in mammalian neurodegeneration [34]. Recent work by Calixto and colleagues showed that in *mec-4(d)* neuronal death Reactive Oxygen Species (ROS) become elevated and that neurons are spared when animals enter in diapause. In this case, neuronal protection is mediated by inhibition of the insulin/IGF-1-like signaling pathway, which leads to activation of protective superoxide dismutases and catalases via *daf-16*/FOXO transcription factor [35]. We thus wondered whether sirtuins, which link nutrient availability to intracellular pathways, might be involved in neuronal protection.

Thus, in this work we used both the *Caenorhabditis elegans* model of *mec-4(d)*-induced neuronal death and of chemical ischemia with azide at low pH [36–38] to investigate the role of mitochondrial sirtuin *sir-2*.*3* in neuronal death [39]. Of the 4 worm sirtuins we focused on *sir-2*.*3* because pilot experiments suggested effects of its knock-out on neuronal death. The other 3 sirtuins, including the highly homologous *sir-2*.*2* (75% identical to *sir-2*.*3*), also localized in the mitochondria, remain untested in these models. We found that knock-out of *sir-2*.*3* protected neurons against both *mec-4(d)* and azide induced neuronal death. Furthermore, we found that treatment with 2-deoxyglucose (2-DG), which blocks glycolysis, further increased protection and that a null mutation in *daf-16*/FOXO transcription factor eliminated protection mediated by knock-out of *sir-2*.*3*. Finally, our data show that ROS becomes elevated in *sir-2*.*3* knock-out animals in chemical ischemia under dietary deprivation (DD) but to a lesser extent than in wild type suggesting more robust activation of ROS scavengers in this mutant. Our work highlights the role of mitochondrial sirtuins in neuronal death and suggests that their knock-out provides protection via *daf-16*/FOXO transcription factor and ROS scavenging. These results add to the growing list of reports supporting that inhibition of certain sirtuns is protective in at least some models of degeneration.

## Results

### Knock-out of a mitochondrial sirtuin is protective against chemically-induced ischemic neuronal death

**Fig 1:**
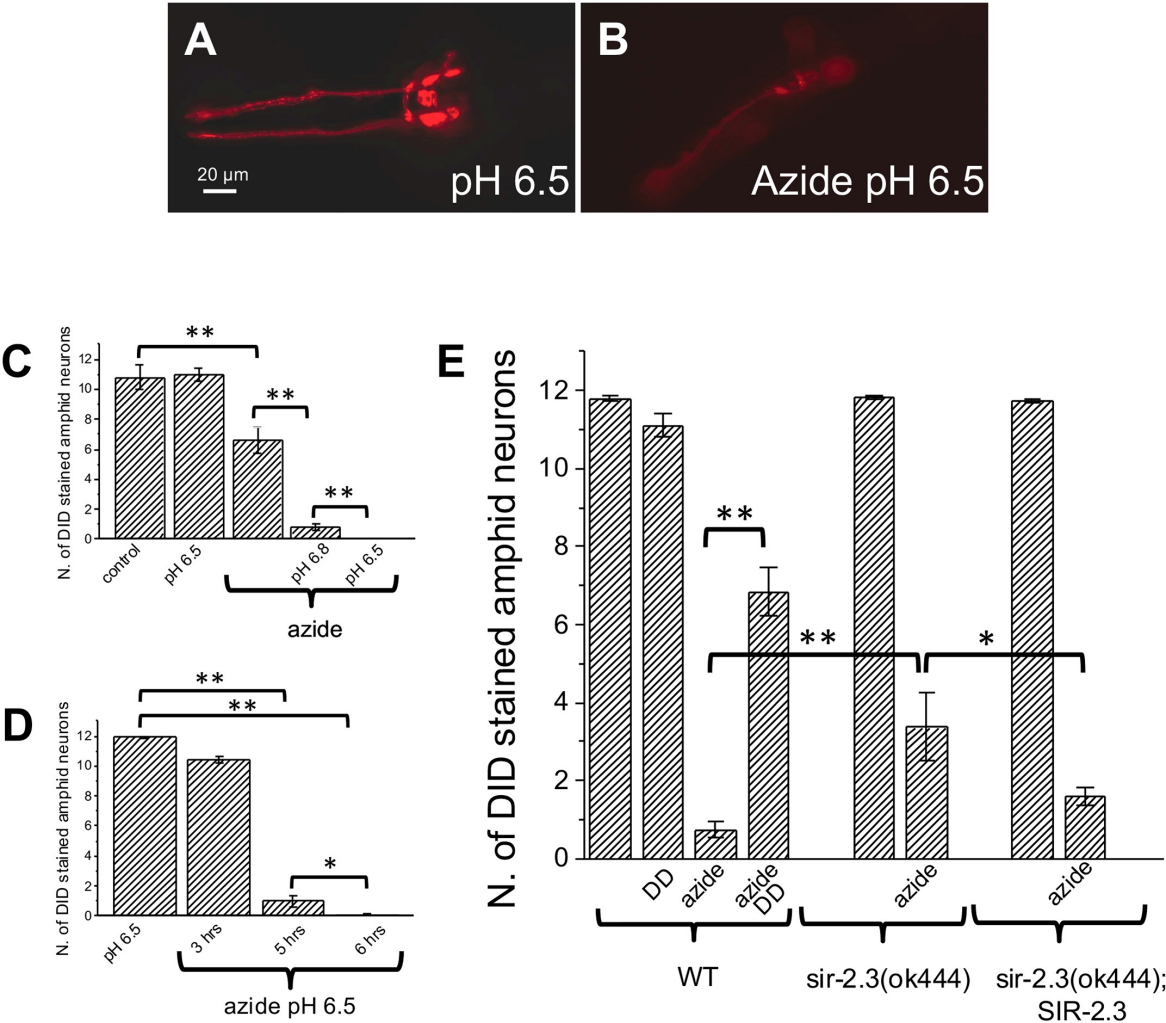
*Sir-2*.*3* knock-out is protective in a model of hypoxic ischemia. **A:** Photograph of the head of a wild type *Caenorhabditis elegans* incubated in S Basal at pH 6.5 for 5 hours and stained with the lipophilic dye DiD. DiD is taken up by 6 pairs of amphid sensory neurons (ASKs, ADLs, ASIs, ASHs, ASJs, and AWBs) exposed to the outside environment. **B:** Photograph of an animal treated with 100 mM azide at pH 6.5 for 5 hours and stained with DiD. Fewer stained sensory neurons are visible in this animal as a result of neuronal death. Scale bar is 20 μm. **C:** Number of DiD stained amphid neurons in wild type animals incubated for 8 hours in S basal pH 7.4 (control), S basal pH 6.5, S Basal pH 7.4 containing 20 mM Na-Azide, S basal at pH 6.8 and pH 6.5 plus 20 mM Na-Azide. N was of experiments was 2 with at least 12 animals for each condition. Data are expressed as mean +/- SE with P values 0.000026, 8.25E-25 and 0.003 by ANOVA with Bonferroni correction. **D:** Number of DiD labeled amphid neurons in wild type treated with S basal pH 6.5 and S basal pH 6.5 plus 100 mM Na-Azide for 3, 5 and 6 hours respectively. N of animals was 12 per condition. Data are expressed as mean +/- SE with P values 1.32E-52, 1.25E-80 and 0.037 by ANOVA with Bonferroni correction. **E:** Number DiD stained amphid sensory neurons in wild type, wild type under dietary deprivation (DD), *sir-2*.*3* knock-out animals, and *sir-2*.*3*;SIR-2.3 rescue animals incubated for 5 hours in S Basal at pH 6.5 and in S Basal at pH 6.5 containing 100 mM Na-azide. At least 50 animals were analyzed in each of 4 separate experiments. Data are expressed as mean +/- SE with P values 0.01, 0.0015 and 0.0002 by ANOVA with Bonferroni correction.

Chemical ischemia was induced by incubation of *Caenorhabditis elegans* in azide [36,40]. Azide blocks complex IV and V of the electron transport chain, blocking ATP production and consequently causing cell death by necrosis [41]. To confirm that this protocol was effective in inducing neuronal death, we stained treated animals with the lipophilic dye DiD. DiD is taken up by neurons that are exposed to the outside environment, including amphid sensory neurons. The uptake of the fluorescent dye requires retrograde transport and therefore it does not occur in dead or sick neurons [42]. To more closely mimic ischemic conditions we chose to dissolve azide in low pH buffer [43–46]. Animals exposed to buffer at pH 6.5 alone show no significant sign of neurons loss (Fig 1A and 1C first 2 columns, 1D and 1E first column from left). In animals exposed to azide alone, 50% of the neurons are stained with DiD (6.6 +/- 0.88) (Fig 1C third column from left). Conversely, animals treated with azide at pH 6.8 and 6.5 show a significantly more pronounced loss of neuronal dye uptake (0.81 +/- 0.21 and 0 respectively) (Fig 1C fourth and fifth column from left). Importantly this effect is time dependent as shown for azide at pH 6.5 at 3, 5, and 6 hours of treatment (Fig 1D, 10.41 +/- 0.2, 1 +/- 0.39 and 0.08 +/- 0.08 respectively). These data show that azide at low pH is more toxic than azide alone, similarly to the demonstrated deleterious effect on neurons of low pH in ischemic conditions [47–49]. We thus chose to use azide at pH 6.5 to induce chemical ischemia in wild type and *sir-2*.*3* knock-out (*sir-2*.*3(ok444)*) [50].

Surprisingly, we found that a significantly higher number of amphid sensory neurons survived in *sir-2*.*3* knock-out mutants versus wild type (3.36 +/- 0.87 and 0.74 +/- 0.21 for *sir-2*.*3* knock-out and wild type respectively) (Fig 1E). Consistently, fewer *sir-2*.*3* knock-out animals die when treated with azide (0.65 +/- 0.08 and 0.33 +/- 0.12 proportion of animals dead for wild type and *sir-2*.*3* knock-out animals respectively, 3 experiments with at least 50 animals per experiment, ** indicates p≤0.01 by t-Test). To confirm that the effects of *sir-2*.*3* knock-out are mediated by the lack of this gene, we quantified amphid sensory neurons DiD uptake in rescue animals in which sir*-2*.*3* was reintroduced under the control of its own promoter in the *sir-2*.*3* knock-out background. As expected for a gene specific effect, the number of amphid sensory neurons that are stained is restored to typical levels found in wild type background in *sir-2*.*3*;SIR-2.3 animals (1.57 +/- 0.43, Fig 1E).

To conclude, these data show that knock-out of mitochondrial sirtuin *sir-2*.*3* in *Caenorhabditis elegans* is protective against cell death caused by chemical ischemia, a surprising result given that activation of sirtuins has been previously suggested to provide protection against ageing and degenerative conditions [3–7].

### Knock-out of *sir-2*.*3* is protective in hyperactive MEC channel induced neuronal death

**Fig 2:**
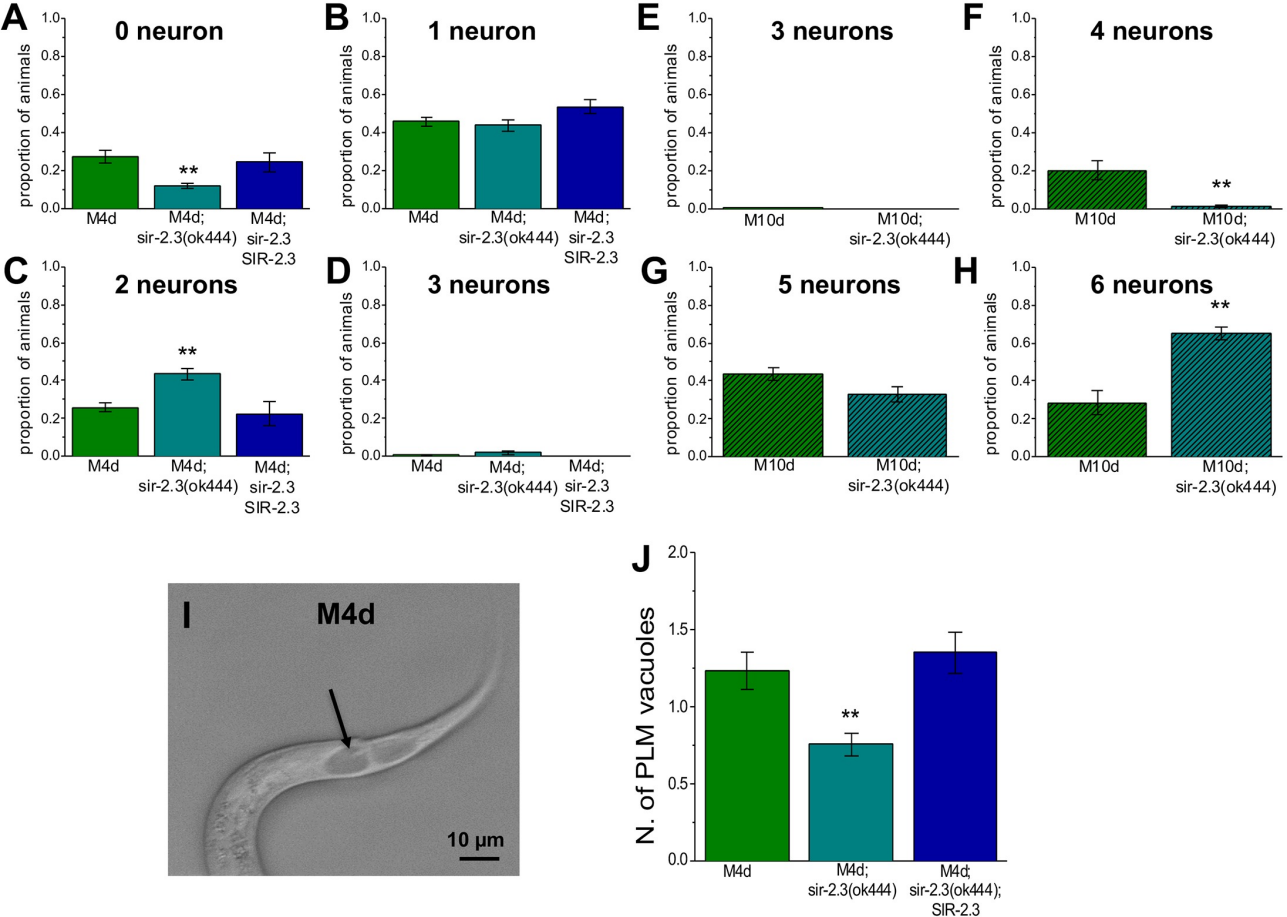
Knock-out of mitochondrial *sir-2*.*3* protects against *mec-4(d)* and *mec-10(d)*-induced neuronal death. **A-D:** Quantification of the proportion of animals with 0, 1, 2 and 3 surviving touch neurons in *mec-4(d)*, *mec-4(d);sir-2*.*3* knock-out and *mec-4(d);sir-2*.*3*;SIR-2.3 rescue strains. Number of experiments was 23, 23, and 4, respectively, with at least 50 animals per strain analyzed in each experiment. P was 0.0002 and 0.0001 by ANOVA with Bonferroni correction. **E-H:** Proportion of *mec-10(d)* expressing animals with 3, 4, 5 and 6 surviving touch neurons. Number of experiments was 4 each, with at least 50 animals per strain analyzed in each experiment. P was 0.01 and 0.0024 by t-Test. **I:** Photograph of the tail of an L1 *mec-4(d)* mutant *Caenorhabditis elegans* showing a necrotic swollen PLM touch neuron, visible as a large vacuole. Scale bar is 10 μm. **J:** Quantification of the number of swollen PLMs in L1 *mec-4(d)*, *mec-4(d);sir-2*.*3* knock-out and *mec-4(d);sir-2*.*3*;SIR-2.3 rescue strains. Number of experiments was 5, 5, and 4, respectively with at least 50 animals per strain analyzed in each experiment. Data are expressed as mean +/- SE with P value 0.014 by ANOVA with Bonferroni correction.

To test whether knock-out of *sir-2*.*3* protects against necrosis induced by hyperactive MEC channel, we crossed *sir-2*.*3* knock-out with *mec-4(d)* and *mec-10(d)* strains and quantified touch neurons degeneration in L4 larvae. MEC-10 is 53% identical to MEC-4 with which it co-assembles to form a heteromultimeric channel complex in *Caenorhabditis elegans* touch neurons [51]. While MEC-4 is the main channel subunit, MEC-10 functions as a modulatory subunit [28,29,51,52]. In line with its modulatory role in channel function, MEC-10 causes only mild degeneration when hyperactivated by mutation A673T corresponding to A713V/T in MEC-4 [51,52]. Consistent with the results in animals treated with azide (Fig 1E), more touch neurons survived in *sir-2*.*3* knock-out animals than they did in animals in which *sir-2*.*3* gene is wild type, when either MEC-4 or MEC-10 were hyperactivated [27,52]. More specifically, the proportion of *mec-4(d)* animals that have no surviving and 2 surviving touch neurons went from 0.27 +/- 0.03 and 0.25 +/- 0.02, to 0.12 +/- 0.01 and 0.43 +/- 0.03 respectively in *sir-2*.*3* knock-out animals, while the proportion of animals with 1 surviving touch neuron remained unchanged. The effect is gene-specific, as the extent of neuronal death is restored to control levels in *mec-4(d);sir-2*.*3*;SIR-2.3 rescue strain (proportion of animals with 0 and 2 surviving touch neurons was 0.24 +/- 0.05 and 0.22 +/- 0.06 respectively, Fig 2A–2D). Similarly, the proportion of *mec-10(d)* animals that have 4 and 6 surviving touch neurons went from 0.20 +/- 0.05 and 0.28 +/- 0.06, to 0.01 +/- 0.01 and 0.65 +/- 0.03 in *sir-2*.*3* knock-out animals, while no change was observed in the proportion of animals with 5 surviving touch neurons (Fig 2E–2H).

To establish whether knock-out of *sir-2*.*3* affords protection in early development (Fig 2I) [27], we examined touch neurons in L1 larvae. We found that there were fewer swollen PLM touch neurons in *sir-2*.*3* knock-out animals as compared to *mec-4(d)* and *mec-4(d);sir-2*.*3*;SIR-2.3 (0.75 +/- 0.07 versus 1.2 +/- 0.12 in *mec-4(d)* and 1.35 +/- 0.13 in *mec-4(d);sir-2*.*3*;SIR-2.3, respectively, Fig 2J). These results support that the protective effects of knock-out of mitochondrial *sir-2*.*3* are present early on in development suggesting that the mechanisms involved are likely not developmentally regulated. To rule out an effect of the knock-out of *sir-2*.*3* on *mec-4(d)* expression level in touch neurons, which would result in changes to the extent of neuronal death, we quantified fluorescence in touch neurons expressing GFP tagged MEC-4. We found that the knock-out of *sir-2*.*3* does not change the level of expression of MEC-4::GFP transgene (GFP intensity in wild type versus *sir-2*.*3* knock-out was 21038 +/- 1666 and 20311 +/- 1866, n = 35 and 29 respectively). Taken together, these results support that knock-out of *sir-2*.*3* provides protection against necrotic cell death induced by channel hyperactivation.

### Protection mediated by *sir-2*.*3* knock-out is independent of SIRT1 homolog SIR-2.1

**Fig 3:**
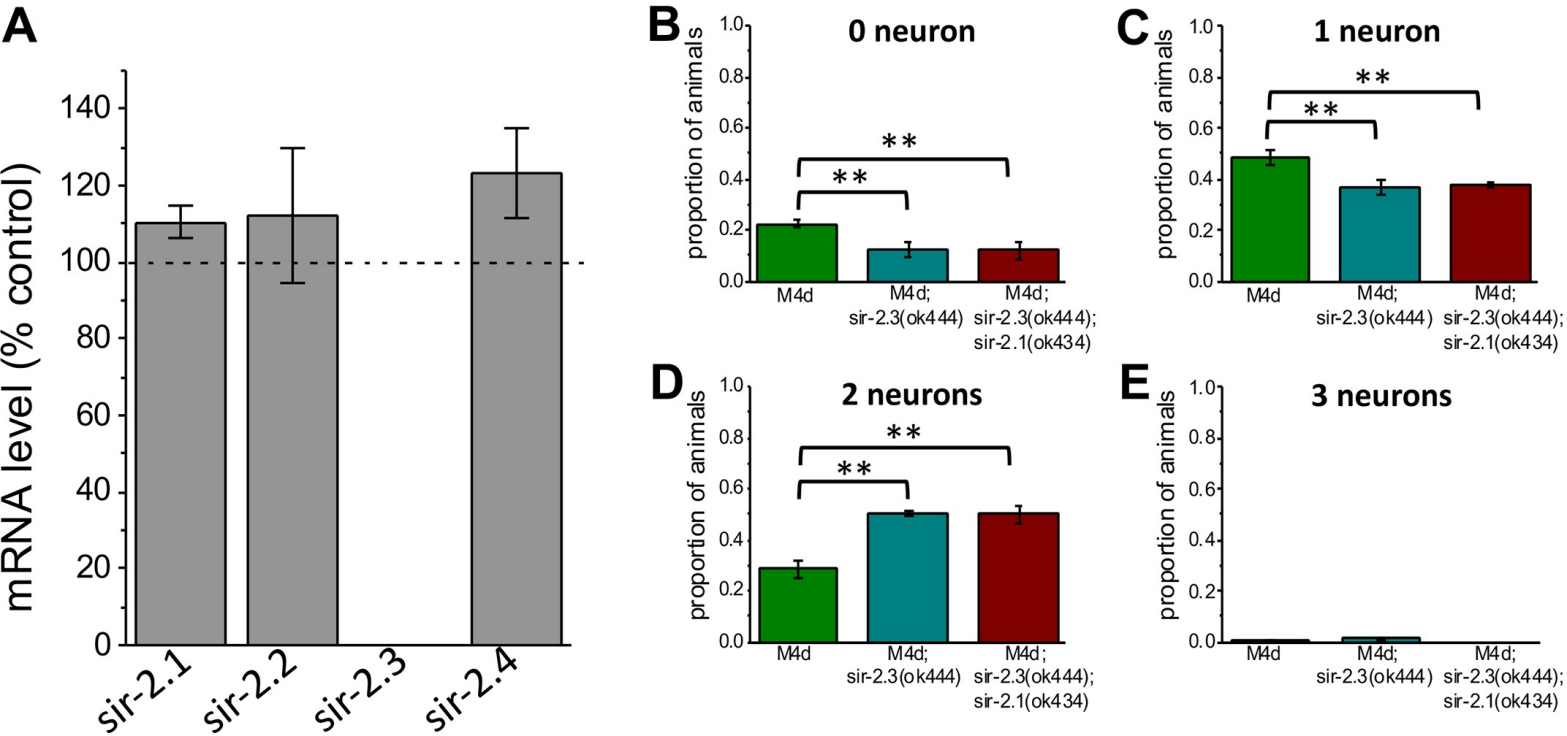
SIR-2.1 does not mediate the protective effects of *sir-2*.*3* knock-out. **A:** Quantitative real-time PCR (qRT-PCR) from total RNA was conducted using Taqman gene expression assays in order to quantify the levels of mRNA for sirtuin genes. mRNA percentage of each target gene was calculated by comparing the cycle threshold of the target gene to that of the housekeeping gene *pmp-3*. The wild-type N2 (Bristol) strain was used as the calibrator. All conditions had three technical replicates. **B-E**: Proportion of animals with 0, 1, 2 and 3 alive touch neurons in *mec-4(d)*, *mec-4(d);sir-2*.*3* and *mec-4(d);sir-2*.*1;sir-2*.*3* mutants. Data are expressed as mean +/- SE, N = 3. P was from left to right and from top to bottom: 0.003, 0.0029, 0.0019, 0.0005, 0.000013 and 0.00009 by ANOVA with Bonferroni correction.

Activation of SIRT1 is protective against ischemia [6,10–13]. To rule out that the protective effect of *sir-2*.*3* knock-out was mediated by compensatory activation of *Caenorhabditis elegans* SIRT1 homolog SIR-2.1, we first examined the mRNA level of sirtuins in *sir-2*.*3* knock-out. We found that the mRNA level of the other 3 sirtuins remained relatively unchanged in *sir-2*.*3* knock-out (Fig 3A). These results support that there is no upregulation of the transcription of other sirtuin genes in *sir-2*.*3* knock-out. Secondly, we examined whether the protective effect of knock-out of *sir-2*.*3* was still present in the absence of the *sir-2*.*1* gene. To do so, we compared touch neurons’ death in *mec-4(d)*, *mec-4(d);sir-2*.*3*, and *mec-4(d);sir-2*.*3;sir-2*.*1* mutants. We found that the extent of neuronal death was similar in *mec-4(d);sir-2*.*3* and *mec-4(d);sir-2*.*3;sir-2*.*1* mutants, supporting that neuronal protection mediated by the knock-out of *sir-2*.*3* is independent of the activity of SIR-2.1 (Fig 3B–3E).

### Block of the nicotinamide adenine dinucleotide (NAD^+^) salvage pathway protects against neuronal death

**Fig 4:**
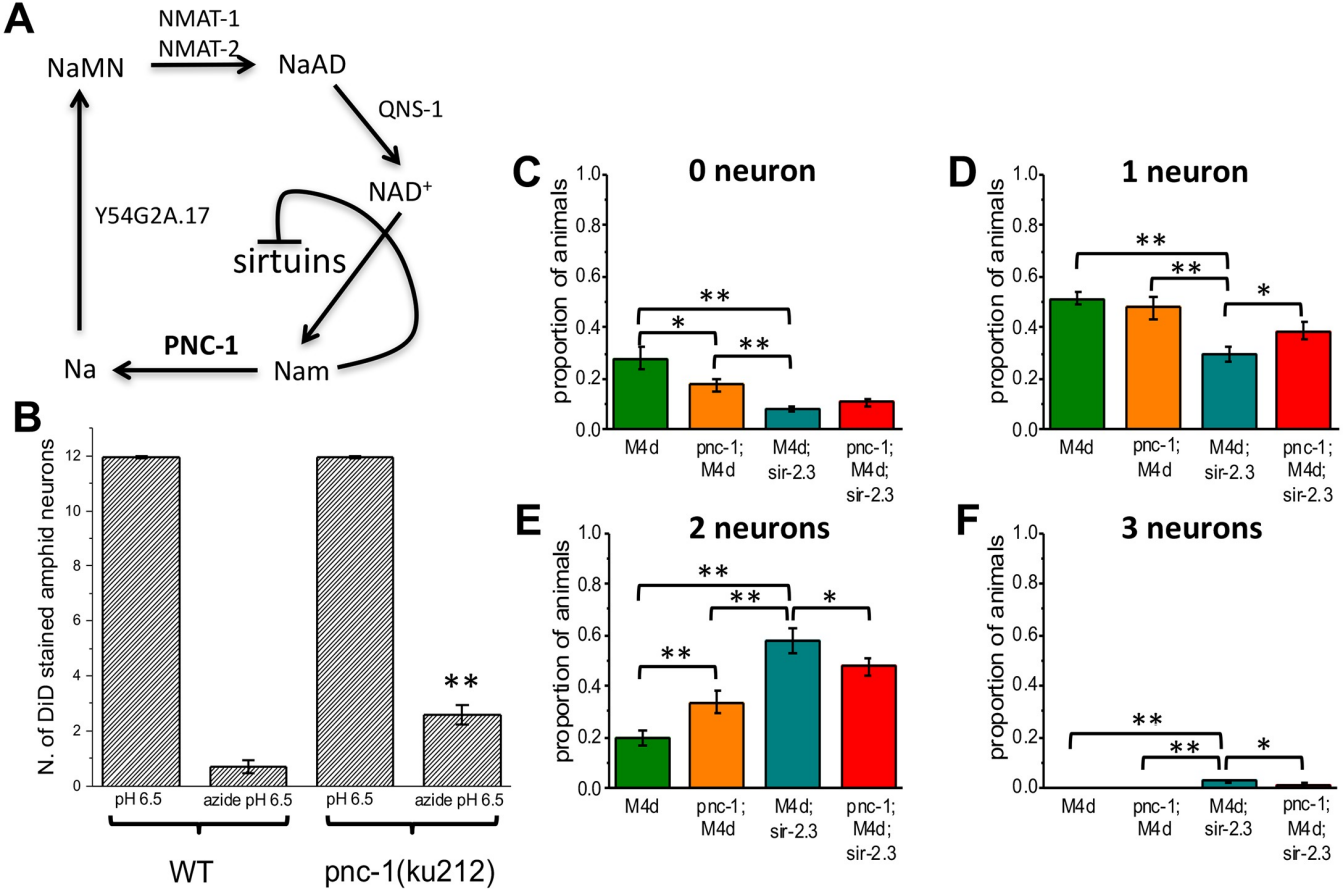
Block of the NAD^+^ salvage pathway by null mutation in pnc-1 protects neurons from hypoxic ischemia. **A:** schematic representation of the invertebrate NAD^+^ salvage pathway. In invertebrates PNC-1 converts nicotinamide into nicotinic acid. A null mutation in *pnc-1* leads to reduced availability of NAD^+^ and accumulation of its substrate nicotinamide, which in turn inhibits the activity of sirtuins. **B**: Average number of DiD stained amphid sensory neurons in WT and *pnc-1(ku212)* mutant in control conditions (pH 6.5) and under conditions of chemical hypoxia (100 mM azide at pH 6.5). Data are expressed as mean +/- SE. Number of animals was 24 and 25 for wild type and 23 and 25 for *pnc-1(ku212)* in control conditions and azide respectively. P was 0.000029 by Student’s t-Test in which the 2 samples in azide were compared with each other. **C-F:** Proportion of animals with 0,1,2 and 3 GFP touch neurons in *mec-4(d)*, *mec-4(d);pnc-1*, *mec-4(d);sir-2*.*3*, *mec-4(d);sir-2*.*3;pnc-1* mutants. Data are expressed as mean +/- SE. Number of experiment was 7,4,3 and 3 respectively with at least 40 animals per strain analyzed in each experiment. P was from left to right and from top to bottom: 9.9E-8, 0.007, 0.001 (panel C), 3.08E-6, 9.07E-4, 0.011 (panel D), 5.04E-6, 0.0013, 2.43E-4, 0.034 (panel E), 2.76E-5, 2.76E-5, 0.013 (panel F) by ANOVA with Bonferroni correction.

Sirtuins use NAD^+^ as co-substrate in their enzymatic reaction. NAD^+^ is synthesized de novo from tryptophan or by recycling of degraded NAD^+^ products, such as nicotinamide through the salvage pathway (Fig 4A) [53,54]. A key enzyme of the NAD^+^ salvage pathway in invertebrates is the nicotinamidase encoded by the PNC1 gene (*pnc-1* in *Caenorhabditis elegans*), which converts nicotinamide into nicotinic acid [55,56] (Fig 4A). Inhibition or knock-out of *pnc-1* reduces the level of NAD^+^ and increase the concentration of nicotinamide in the cell, as this PNC-1 substrate is no longer metabolized. The end result is inhibition of sirtuins, as nicotinamide functions in a negative feedback loop to inhibit sirtuins function [57].

We thus reasoned that blocking the NAD^+^ salvage pathway may produce a similar effect on neuronal death as the knock-out of *sir-2*.*3*. To test this hypothesis, we acquired the *pnc-1(ku212)* mutant, which encodes a premature stop mutation in both pnc-1a and pnc-1b isoforms leading to complete loss of enzymatic activity [58]. *pnc-1(ku212)* mutants have developmental defects in the reproductive system, including delayed development of the gonad and necrosis of the four uterine cells, and they are egg-laying defective [58,59], but have normal amphid sensory neurons. When we quantified the number of amphid sensory neurons that uptake DiD in *pnc-1(uk212)* animals treated with azide, we found that they were more than in wild type (2.58 +/- 0.34 versus 0.68 +/- 0.23) (Fig 4B), suggesting that reduced NAD^+^ or/and increased nicotinamide in *pnc-1(ku212)* might inhibit sirtuins, consistent with data shown in Figs 1 and 2. To further test this idea we turned again to the *mec-4(d)* model and crossed *pnc-1* mutant with both *mec-4(d)* and *mec-4(d);sir-2*.*3* mutants. When we compared the number of surviving touch neurons in these genetic backgrounds we found that, like in animals treated with azide, some protection was observed in *pnc-1* mutant (proportion of animals with 0, 1, and 2 surviving touch neurons was 0.28 +/- 0.04, 0.51 +/- 0.02 and 0.19 +/- 0.03 respectively in *mec-4(d)* and 0.17 +/- 0.02, 0.47 +/- 0.04 and 0.33 +/- 0.04 respectively in *pnc-1;mec-4(d)*, Fig 4C–4F). But more importantly, we observed that the effects on neuronal protection seen of *sir-2*.*3* knock-out and *pnc-1* mutant are not additive suggesting a common genetic pathway (proportion of animals with 0, 1, 2 and 3 surviving touch neurons was 0.08 +/- 0.01, 0.29 +/- 0.028, 0.57 +/- 0.04 and 0.02 +/- 0.005 respectively in *mec-4(d);sir-2*.*3* and 0.11 +/- 0.01, 0.38 +/- 0.03, 0.48 +/- 0.03 and 0.011 +/- 0.005 respectively in *pnc-1;mec-4(d);sir-2*.*3*, Fig 4C–4F). To conclude results obtained in *pnc-1* mutants support our hypothesis that protection from neuronal death can be induced by inhibition of sirtuins. An effect on glycolysis of *pnc-1(ku212)* mutation is less likely to explain the effects observed [60], consistent with data shown in Fig 1E (4^th^ column from the left) and with data shown by Calixto and colleagues [35].

### Block of glucose metabolism reduces *mec-4(d)* induced neuronal death

**Fig 5:**
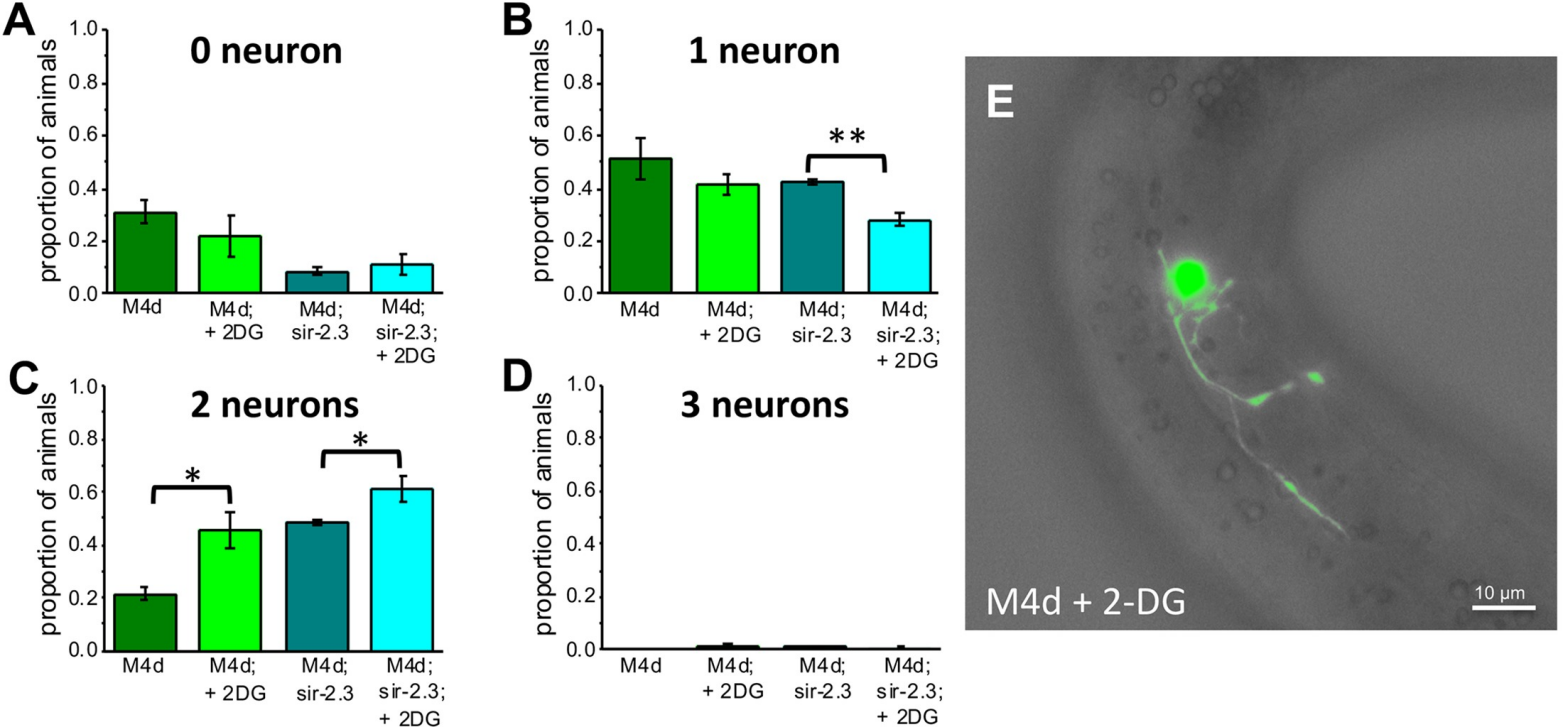
Protection afforded by block of glycolysis in the presence and absence of SIR-2.3. **A-D:** Proportion of animals with 0, 1, 2, or 3 surviving touch neurons that were either grown on standard plates or on plates containing 5 mM 2-DG for 48 hours. Number of experiments was 4 each, with at least 30 animals in each experiment. Data are expressed as mean +/- SE. P was 0.0012, 0.018 and 0.048, by t-Test. **E:** Photograph of an ALM touch neuron in a *mec-4(d)* animals grown on 2-DG plate. The neuronal processes are still intact and show some arborization. Scale bar is 10 μm.

To learn more about the role of glycolysis in the mechanism underlying the protective effect mediated by *sir-2*.*3* knock-out, we used 2-deoxyglucose. 2-deoxyglucose (2-DG) is a glucose analogue that is taken up by the glucose transporters and is phosphorylated but cannot be fully metabolized, causing inhibition of glycolytic enzymes. We cultured *mec-4(d)* worms on plates containing 5 mM 2-DG and found that neuronal death is reduced (proportion of animals with 0 and 2 surviving touch neurons went from 0.31 +/- 0.04 and 0.21 +/- 0.03 for animals cultured on standard plates to 0.22 +/- 0.08 and 0.45 +/- 0.07 for animals cultured on 2-DG plates respectively) (Fig 5A–5D). Importantly, surviving neurons appeared also healthier as they retained at least part of their neuronal processes (Fig 5E). Thus, block of glycolysis provides protection against *mec-4(d)-*induced neuronal death.

We next wondered whether the protection afforded by 2-DG treatment and knock-out of *sir-2*.*3* are mediated by the same mechanisms. To test this possibility, we treated *mec-4(d);sir-2*.*3* mutants with 2-DG and compared the extent of cell death with untreated animals and with *mec-4(d)* animals treated with 2-DG. We found that 2-DG enhances protection in *mec-4(d);sir-2*.*3* beyond the level seen in untreated *mec-4(d);sir-2*.*3* animals and in *mec-4(d)* animals treated with 2-DG (proportion of animals with 0, 1 and 2 surviving touch neurons in *mec-4(d);sir-2*.*3* was 0.11 +/- 0.02, 0.33 +/- 0.04 and 0.54 +/- 0.05 respectively Fig 5A–5D). These results show that 2-DG and knock-out of *sir-2*.*3* mediate protection through two different mechanisms and further support that the effects on neuronal death seen in *pnc-1* mutant (Fig 4) are independent from glycolysis and likely mediated by reduction of NAD^+^ and accumulation of nicotinamide in the cell in this mutant.

### Analysis of neuronal death in culture

**Fig 6:**
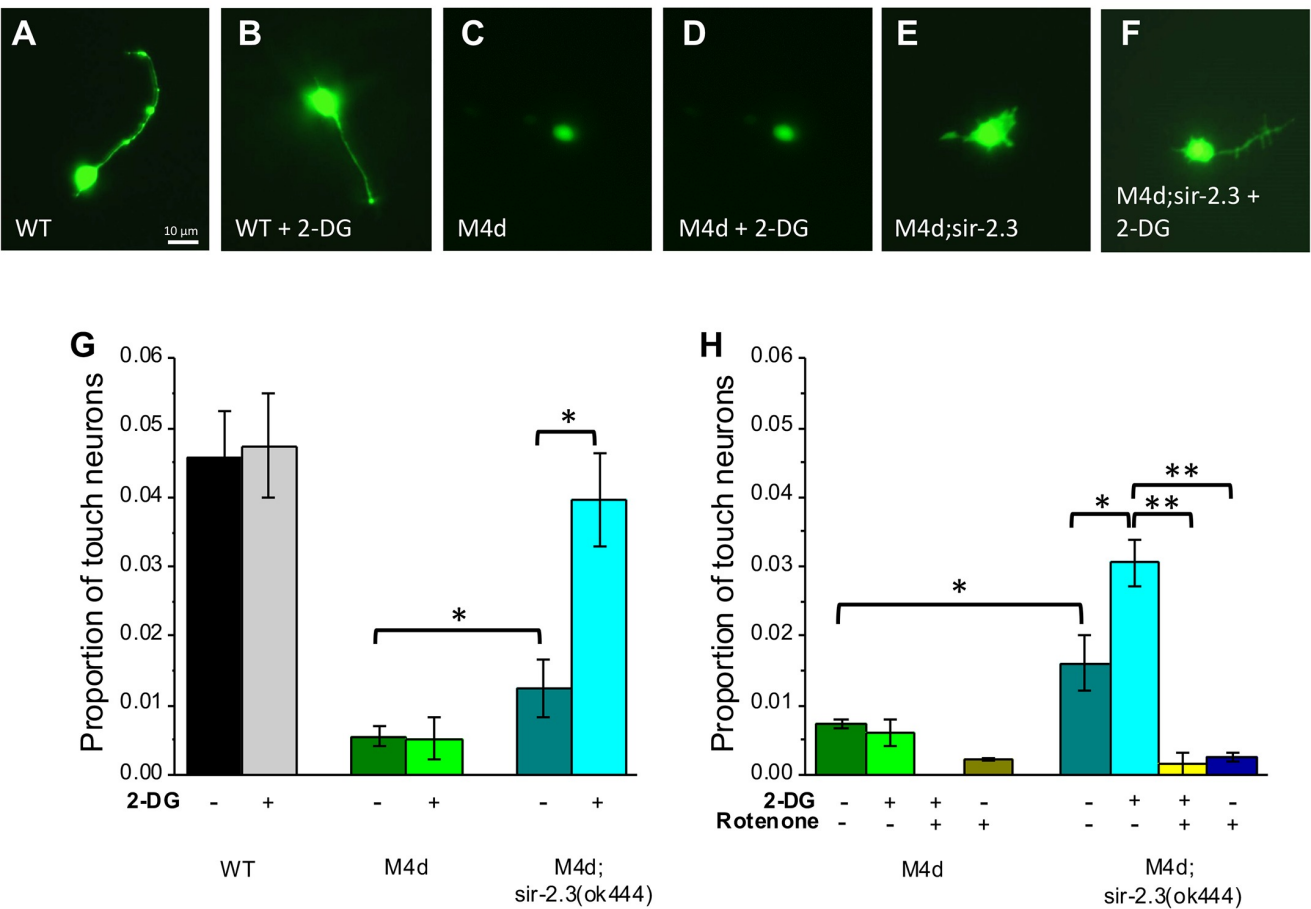
Effect of block of glycolysis and knock-out of *sir-2*.*3* on *mec-4(d)*-induced neuronal death in cultured cells. **A-F:** Fluorescent images of wild type, *mec-4(d)*, and *sir-2*.*3;mec-4(d)* touch neurons expressing P_mec-4_::GFP cultured *in vitro*. Cells were in control media (A,C,E) or in media containing 10 mM 2-deoxy-glucose(2-DG)(B,D,F). Scale bar is 10 μm. **G:** Quantification of surviving touch neurons in culture for the genetic strains shown in the micrographs, in control media and in media containing 10 mM 2-DG. Data are expressed as the means +/- SE. Similar results were obtained in 5 independently performed experiments. N is 2 coverslips for each strain (wild type, *mec-4(d)* and *sir-2*.*3;mec-4(d)*) and for both conditions (control and 10 mM 2-DG respectively), with at least 10 fields scored per coverslip. **H:** Quantification of surviving touch neurons in culture for an experiment similar to the one shown in panel G for *mec-4(d)* and *sir-2*.*3;mec-4(d)*. In this case the culturing conditions were: control, 10 mM 2-DG, 2.5 μM rotenone and 10 mM 2-DG + 2.5 μM rotenone. N is 2 coverslips each with at least 10 fields scored per coverslip. P was 0.039 and 0.015 (panel G) and 0.019, 0.007, 0.00007 and 0.00006 (panel H) by ANOVA with Bonferroni correction.

*Caenorhabditis elegans* embryonic cells, including touch neurons, can be dissociated and cultured in *in vitro* where they differentiate and express cell specific markers [29,61,62]. We cultured *Caenorhabditis elegans* embryonic cells from wild type, *mec-4(d)*, and *mec-4(d);sir-2*.*3* both in control conditions and in the presence of 2-DG. Touch neurons were labeled by expression of GFP under the control of the *mec-4* promoter (Fig 6A–6F). As expected, we found that touch neurons make up ~5% of the embryonic cells, elongate one single neuronal process, and express GFP under the control of the *mec-4* promoter. Treatment with 2-DG does not change wild type touch neurons proportion in the cell population or their morphology (Fig 6A, 6B and 6G). As previously published, we found that *mec-4(d)* touch neurons undergo degeneration in cell culture [29]. The rarely surviving *mec-4(d)* touch neurons do not have neuronal processes (Fig 6C, 6D and 6G). Surprisingly, addition of 2-DG to *mec-4(d)* touch neurons does not rescue them from degeneration (Fig 6G). These data are in contrast with *in vivo* results (Fig 5) and suggest that protection does not occur in culture, perhaps due to hyperactivation of the insulin/IGF pathway by insulin peptides present in the culture media.

Interestingly though, and in line with the results of *in vivo* experiments (Fig 5A–5D), knock-out of *sir-2*.*3* does protect neurons from neuronal death in culture also and this protective effect is further enhanced by treatment with 2-DG. These results suggest that the rescue of neuronal death afforded by *sir-2*.*3* knock-out is mediated by a mechanism that differs from the insulin/IGF pathway or that knock-out of *sir-2*.*3* bypasses the DAF-2/IGF-1 receptor warranting protection *in vitro* as well. Finally, we found that the protective effect of *sir-2*.*3* knock-out and of 2-DG in this mutant was completely abolished by treatment with rotenone, a blocker of the mitochondrial complex I, suggesting the requirement of mitochondrial function for protection to be carried out (Fig 6H).

### DAF-16/FOXO transcription factor is required for protection against neuronal death mediated by knock-out of *sir-2*.*3*

**Fig 7:**
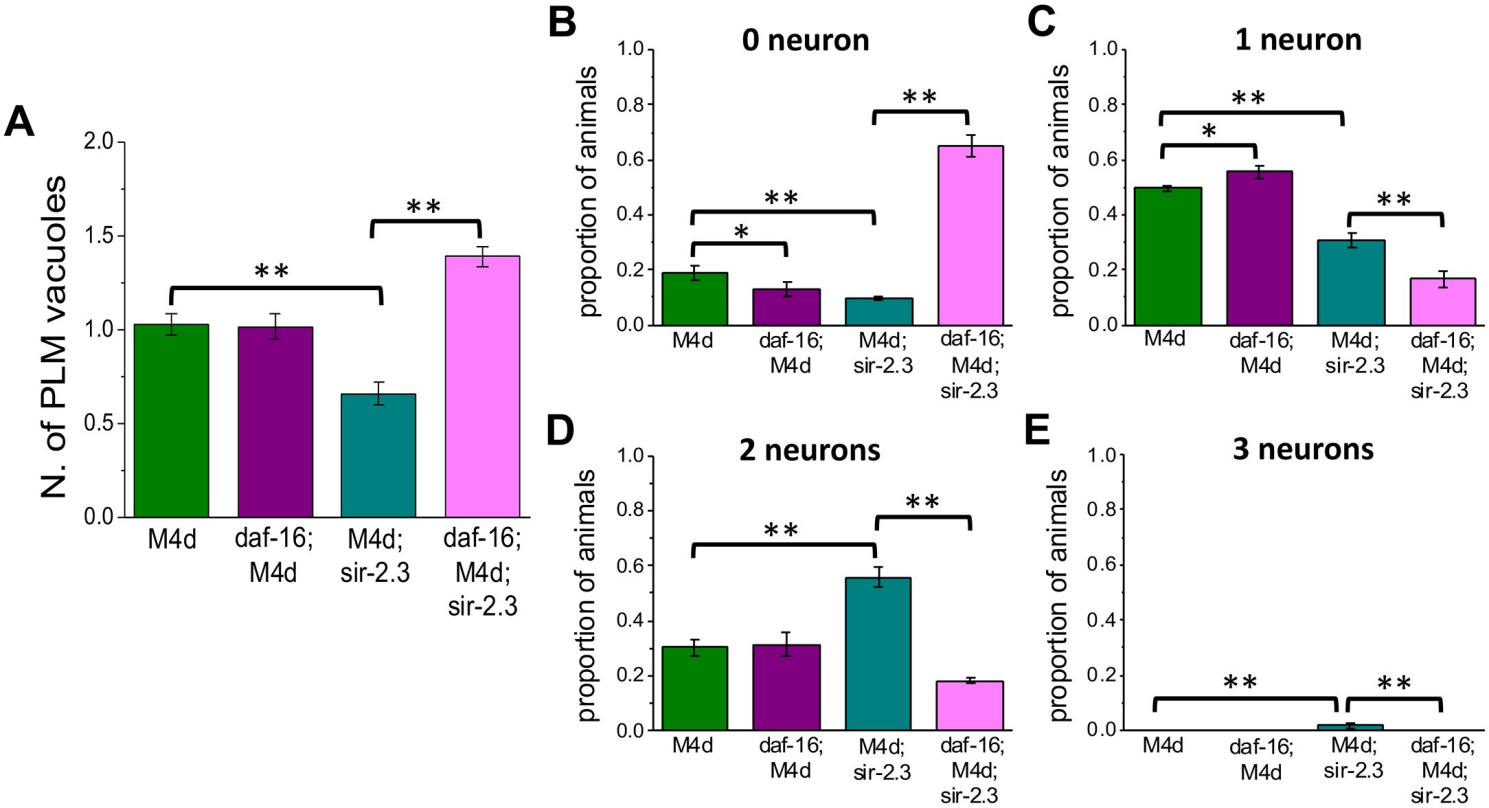
Neuronal protection mediated by *sir-2*.*3* knock-out depends on daf-16/FOXO transcription factor. **A:** Number of vacuolated PLM neurons in L1 *mec-4(d)*, *daf-16;mec-4(d)*, *mec-4(d);sir-2*.*3*, and *daf-16*;*mec-4(d);sir-2*.*3* mutants. Number of experiments is 3 each with at least 40 animals per strain analyzed in each experiment. Data are expressed as mean +/- SE. P was 1.96E-9 and 2.82E-33 by ANOVA with Bonferroni correction. **B-E:** Proportion of animals with 0, 1, 2 and 3 surviving touch neurons in *mec-4(d)*, *daf-16;mec-4(d);*, *mec-4(d);sir-2*.*3* and *daf-16;mec-4(d);sir-2*.*3* mutants. Number of experiments was 3 each, with at least 45 animals per strain analyzed in each experiment. P was from left to right and from top to bottom: 0.030, 0.00045, 5.43E-9, 0.006, 2.57E-6, 0.000416, 2.84E-5, 2.01E-7, 0.027 and 0.027 by ANOVA with Bonferroni correction.

To distinguish between the involvement of the insulin pathway downstream of the DAF-2/IGF-1 receptor and an entirely different mechanism underlying protection mediated by *sir-2*.*3* knock-out, we tested the effect of a null mutation in *daf-16* (*daf-16(mu86)*) on MEC channel induced necrosis in *sir-2*.*3* knock-out animals. To do so, we crossed *daf-16(mu86)* with *mec-4(d)* and *mec-4(d);sir-2*.*3* strains and counted PLM vacuoles and GFP labeled touch neurons in L1 and L4 larvae respectively. We found that lack of transcription factor DAF-16 prevented protection mediated by knock-out of *sir-2*.*3* evidenced by the higher number of PLM vacuoles in L1 (1.38 +/- 0.052 in *daf-16;mec-4(d);sir-2*.*3* versus 0.65 +/- 0.05 in *mec-4(d);sir-2*.*3*, Fig 7A) and the fewer number of alive touch neurons in L4 in *daf-16;mec-4(d);sir-2*.*3* versus *mec-4(d);sir-2*.*3* animals (proportion of animals with 0, 1, 2 and 3 touch neurons alive was 0.097 +/- 0.007, 0.31 +/- 0.02, 0.56 +/- 0.04, and 0.016 +/- 0.01 in *mec-4(d);sir-2*.*3* animals and 0.65 +/- 0.038, 0.16 +/- 0.03, 0.18 +/- 0.01, and 0 +/- 0 in *daf-16*;*mec-4(d);sir-2*.*3*, Fig 7B–7E). Lack of DAF-16 even further worsened the cell death phenotype causing more touch neurons to die as compared to the parental *mec-4(d)* strain. These data support that the insulin pathway is involved in the mechanism of protection mediated by knock-out of *sir-2*.*3* and even suggest that DAF-16 is more involved in keeping neurons alive in *sir-2*.*3* than it is in the *mec-4(d)* strain. We speculate that the results of the cell culture experiments (Fig 6) indicate that nuclear translocation of DAF-16 in *sir-2*.*3* knock-out occurs even in conditions in which DAF-2 is likely stimulated by ligands present in the culturing media, suggesting an effect of *sir-2*.*3* knock-out downstream and independent of the DAF-2 receptor. Future experiments will determine the targets of *sir-2*.*3* that mediate these effects when this mitochondrial sirtuin is knocked-out.

### Elevation of ROS and protection against neuronal death

**Fig 8:**
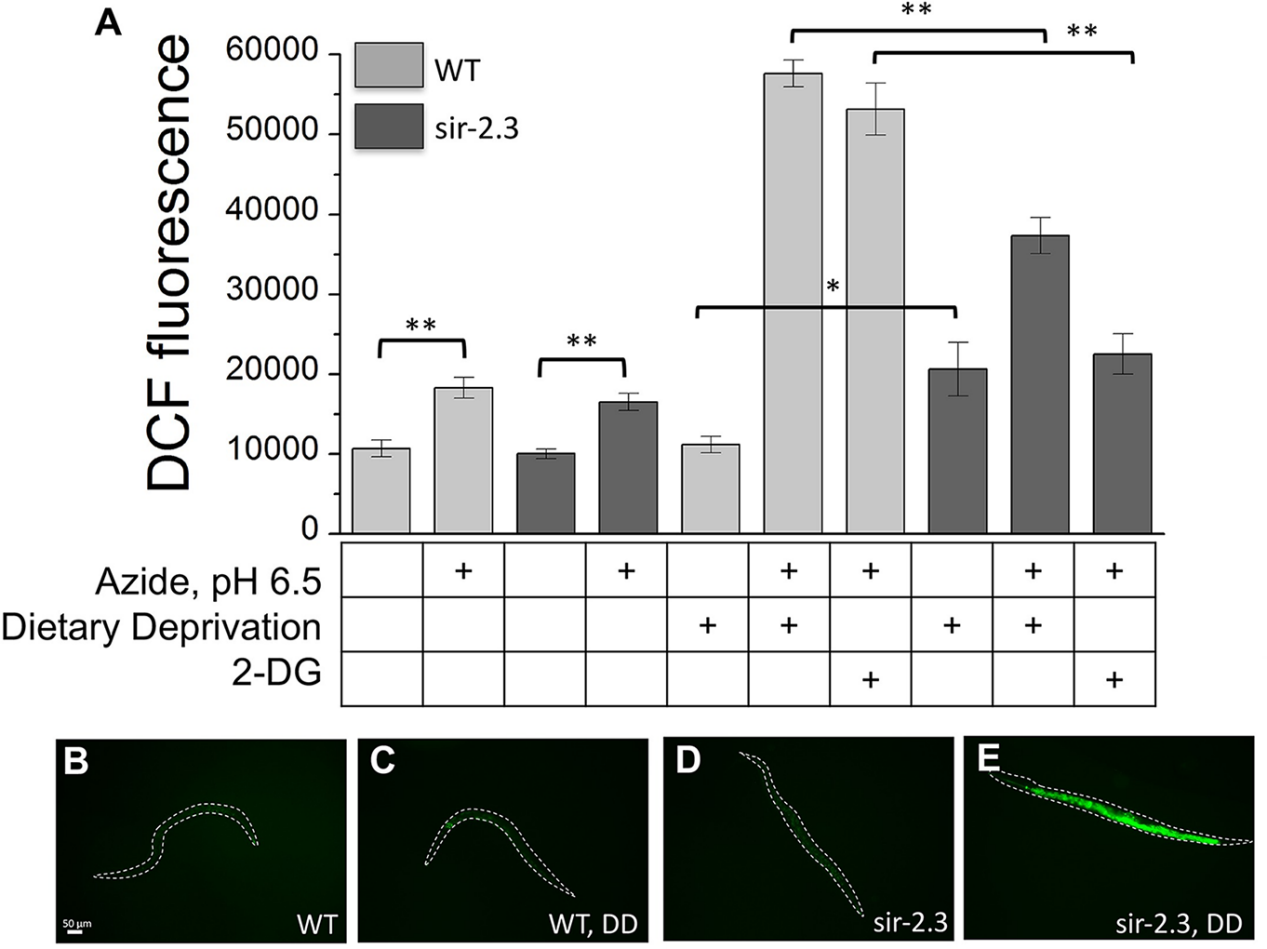
ROS in *sir-2*.*3* knock-out animals. **A:** Quantification of DCF fluorescence in wild type and *sir-2*.*3* knock-out mutants grown under standard condition, dietary deprivation (DD), or on plates containing 5 mM 2-DG for 48 hours with or without treatment with 100 mM Na-azide for 2 hours. Data are expressed as mean +/- SE, P was (from left to right) 0.0017, 0.0068, 0.042, 0.00001, and 0.00001 by ANOVA with Bonferroni correction. N was (from left to right) 22, 55, 23, 68, 25, 23, 23, 16, 38 and 35. **B-E:** Representative photographs of wild type and *sir-2*.*3* mutant animals grown under standard condition (B and C) or in dietary deprivation (DD) for 48 hours (D and E) and stained with the fluorescent ROS indicator DCF. The dotted lines correspond to the animal contour. Scale bar is 50 μm.

Diapause entry mediates protection by activation of superoxide dismutases and catalases via the insulin/IGF-1-like signaling pathway [35]. Since knock-out of *sir-2*.*3* mediates protection via *daf-16*/FOXO we tested whether ROS scavenging was more robust in this mutant under ischemic conditions. We thus compared staining with DCF, which detects H2O2, of wild type and *sir-2*.*3* knock-out animals after 2 hours treatment with 100 mM azide. We found that under ischemic insult ROS becomes elevated in the two strains to a similar extent, ruling out a more effective ROS scavenging in *sir-2*.*3* knock-out as compared to wild type in the presence of food (Fig 8A).

However, when we analyzed DCF staining under ischemic conditions in animals under DD or cultured in the presence of 2-DG, we found that while ROS was 6 times more elevated in wild type animals, it was only 3 to 4 times elevated in *sir-2*.*3* knock-out animals. These data suggest that ROS scavenging is more robust in *sir-2*.*3* knock-out under DD or in conditions in which glycolysis is blocked (Fig 8A). Interestingly, we observed a moderate (about 2 times) elevation of ROS in *sir-2*.*3* knock-out animals in DD (Fig 8A, 8^th^ column from the left). We interpret this result to suggest that DD might induce mitormethic increase of ROS in *sir-2*.*3* mutants [63,64], which in turn activates protective mechanisms during the ischemic insult. Thus analysis of ROS levels suggest that while under DD and in conditions in which glycolysis is blocked mitohormesis might explain the protection against ischemic insult that we observe in *sir-2*.*3* knock-out animals, in well-fed animals another mechanism mediated by *daf-16*/FOXO is responsible for mediating protection.

## Discussion

In this work, we investigated the role of *Caenorhabditis elegans* mitochondrial sirtuin SIR-2.3, homologous to mammalian SIRT4, in two distinct models of neuronal death with morphological and molecular features of necrosis that occurs in cerebral ischemia. We showed that knock-out of *sir-2*.*3* is protective against neuronal death in both models, both under severe and mild toxic insults, and that protection is enhanced by dietary deprivation and block of glycolysis. These results speak of a broad mechanism of neuronal protection. We further showed that this mechanism of protection is cell autonomous and does not require interaction with other cells or secreted molecules. Protection afforded by *sir-2*.*3* knock-out is mediated by *daf-16*/FOXO, but experiments in cell culture suggest that it bypasses the *daf-2*/IGF receptor. We conclude that knock-out of mitochondrial sirtuin *sir-2*.*3* induces changes of mitochondrial or cellular function, that result in more efficient response to toxic insults, suggesting a deleterious role of SIRT4 during the ischemic processes that must to be further investigated.

### Mitochondrial sirtuins, the insulin/IGF-1-like pathway and ROS

We showed that the protective effect of knock-out of *sir-2*.*3* is eliminated by a *daf-16* null mutation, implicating the insulin/IGF-1-like pathway in this mechanism of protection. Calixto and colleagues showed that DAF-16 mediated protection in *mec-4(d)*-induced neuronal death is via activation of superoxide dismutases and catalases that reduce the level of toxic ROS in the cell therefore promoting cell survival [35]. Indeed, in *mec-4(d)*-induced neuronal death, calcium enters the cell through the hyperactivated MEC-4(d) channel, leading to calcium release from the endoplasmic reticulum, mitochondrial dysfunction, and ROS generation [27–29,31,35]. The insulin/IGF-1-like pathway has been involved in several different cellular processes including stress resistance and ageing across species [65] and one of the ways in which it promotes cellular protection is via regulation of anti-oxidant mechanisms [66].

The results in cell culture though (Fig 6) suggest that the protective mechanism activated by knock-out of *sir-2*.*3* and dependent on *daf-16*, may bypass the *daf-2*/IGF-1-like receptor. Indeed we find it peculiar that block of glycolysis has no effect on *mec-4(d)* in cell culture while it enhances neuronal survival *in vivo* (Fig 5 and [35]). Thus, we interpret this result to suggest that insulin like peptides present in the culture media overstimulates the DAF-2 receptor. This is not occurring in *sir-2*.*3* knock-out though, where protection is seen in culture as well (compare Figs 5 and 6) and where it is further enhanced by block of glycolysis. Future studies will determine at what level of the insulin/IGF-1 pathway the modulation by *sir-2*.*3* occurs. Interestingly, SIRT4 is involved in controlling the serotoninergic pathway in mammals [67], which is pivotal in regulating oxidative stress and insulin/IGF-1 signaling pathway in neurons [68].

Our data support that more robust ROS scavenging is occurring in *sir-2*.*3* knock-out under dietary deprivation and when glycolysis is blocked. Furthermore, dietary deprivation alone induces increase of ROS in *sir-2*.*3* knock-out animals (Fig 8). These results point to a mitohormetic effect of ROS in *sir-2*.*3* knock-out animals under dietary deprivation that may explain these experimental results [63,64].

### Insights into the metabolic state of *sir-2*.*3* knock-out animals

A recent report shows drastically reduced resistance to oxidative stress induced by paraquat in *Caenorhabditis elegans* overexpressing *sir-2*.*3*::*gfp* (and *sir-2*.*2*::*gfp*) and only mild reduction of resistance in *sir-2*.*3* knock-out animals [39]. In our study, we did not observe enhanced cell death in rescue animals (Figs 1E and 2), which likely overexpress the *sir-2*.*3* transgene. This discrepancy might be due to the type of toxic insult (paraquat versus azide and *mec-4(d)*); but data from Wirth and colleagues at least suggest a more deleterious effect of overexpression of *sir-2*.*3* versus its knock-out. The same study identified mitochondrial biotin-dependent carboxylases, pyruvate carboxylase (*pyc-1*), propionyl-coenzyme A (−CoA) carboxylase alpha subunit (*pcca-1*), and F32B6.2 (ortholog to human alpha methylcrotonoyl-coenzyme A(−CoA) carboxylase 1 alpha subunit) as proteins that interact with SIR-2.3. Importantly, 1) the interaction is conserved across species as mouse SIRT4 interacts with the same proteins, 2) these carboxylases are heavily acetylated suggesting that SIR-2.3 and SIRT4 may function as deacetylases, and 3) these enzymes play major roles in anaplerosis and energy homeostasis. These published data together with our observations suggest that animals that lack *sir-2*.*3* may be in a different metabolic state that promotes cell survival. This idea is supported by the more significant contribution of DAF-16 to cell survival in *sir-2*.*3* knock-out animals than in control animals (Fig 7).

It is not clear at this point whether SIR-2.3 (or SIRT4) functions as deacetylase. The mitochondrial carboxylases SIR-2.3 and SIRT4 interact with are heavily acetylated but the acetylation level of at least one of them (pyruvate carboxylase) does not change in in the absence of SIRT4 or when SIRT4 is overexpressed. Moreover, SIRT4 does not change its stability or enzymatic activity in control culture conditions and under glucose deprivation [39]. While these results could be due to the experimental conditions (cell culture, compensation mechanisms), these data suggest that the deacetylation substrates of SIRT4 and SIR-2.3 are still unknown. Another possibility is that these mitochondrial sirtuins may have a different enzymatic activity. For example SIRT4 possesses a well-documented ADP-ribosyltransferase activity and one of its targets is the mitochondrial enzyme glutamate dehydrogenase (GDH) [69]. While we do not know whether SIR-2.3 has ADP-ribosyltransferase activity, it is interesting to note that GDH mediates the conversion of glutamate into alpha-ketoglutarate, a Krebs cycle intermediate which acts a ROS scavenger. By mediating ADP-ribosylation of GDH, SIRT4 inhibits the activity of this enzyme. Knock-out of SIRT4 is thus expected to induce increase of the concentration of alpha-ketoglutarate due to disinhibition of GDH, which would increase the anti-oxidant capacity of the cell. In line with this idea, it has been recently shown that direct activation of GDH by activators protects against brain ischemia and reperfusion [70]. Future experiments will establish whether GDH-1 plays a role in neuronal protection mediated by knock-out of *sir-2*.*3*.

To conclude, we have shown here that the knock-out of mitochondrial sirtuin *sir-2*.*3* protects neurons from ischemic damage, especially when paired with dietary deprivation or block of glycolysis. Neuronal protection mediated by knock-out of *sir-2*.*3* requires the *daf-16*/FOXO transcription factor and, under dietary deprivation, may be at least in part mediated by mitohormetic elevation of ROS. Our data also suggest that knock-out of this mitochondrial sirtuin changes the state of the cell so that it becomes more dependent on *daf-16* for survival (Fig 7) and bypasses the *daf-2*/IGF-1 receptor (Fig 5). Our work extends our understanding of mitochondrial situins and of the interplay of glycolysis, Krebs cycle, and respiratory chain in the control of the cellular redox state and survival. Given the parallelism between the models of neuronal death used in this work and ischemia in humans, our work suggests novel approaches targeting SIRT4 and cautions about the use of non-specific sirtuins activators or inhibitors.

## Materials and methods

### Reagents

2-Deoxy-D-Glucose (Sigma-Aldrich, D8375-5G), Sodium Azide (Sigma Aldrich, S8032-25G), DiD solid (Life Technologie, D7757), Leibovitz’s L-15 medium (Life Technologies, 11415–064), Chitinase from Streptomyces Griseus (Sigma, C6137-25 UN), Fetal Bovine Serum (Invitrogen, 16140–063), Penicillin-Streptomycin (Sigma Aldrich, P4333-100ML), Peanut Lectin (Sigma Aldrich, L0881-10MG), Vectashield mounting medium (Vector Laboratories, H-1000), Triazol reagent (Invitrogen, 15596–026), High capacity RNA-to-cDNA Kit (Applied Biosystem, 43874006), Taqman Universal Master Mix II (Applied Biosystem, 4440043),Taqman probe Ce02485188_m1 (Life Technologies, 4448802), Taqman probe Ce02502871_g1 (Life Technologies, 4448892) Taqman probe Ce02502865_g1 (Life Technologies, 4448892) H_2_-DCFDA (Sigma-Aldrich, 35845-1G).

### *Caenorhabditis elegans* strains and growth

Nematodes were kept at 20°C on standard nematode growth medium (NMG) seeded with *Escherichia Coli* (strain OP50^-^) [71] as food source. For experiments with 2-deoxy-glucose (2-DG), animals were grown on plates in which 2-DG was dissolved in the agar to a final concentration of 5 mM. All animals used in this study were hermaphrodites. Males were used for crosses only. Double mutants were generated by standard crosses. Mutations were followed through the crosses by PCR and sequencing.

The following *Caenorhabditis elegans* strains were used in this study: Wild-type N2 Bristol, *zdls5 [pmec-4*::*GFP] I* to label touch neurons with GFP, ZB1656 (*zdls5 [pmec-4*::*GFP] I; mec-4(u231) X*), IS111 (*EX [MEC-10(A673T);pmec-4*::*mcherry*), RB654 (*sir-2*.*3(ok444) X*), BLC231 (*sir-2*.*3(ok444) X; EX[zdls5 (pmec-4*::*GFP) I; mec-4(u231) X]*), BLC320 (*sir-2*.*1(ok434) IV; sir-2*.*3(ok444) X; EX[zdls5 (pmec-4*::*GFP) I; mec-4(u231) X]*), BLC230 (*sir-2*.*3(ok444) X; EX [MEC-10(A673T);pmec-4*::*mcherry) X*::*GFP]*), MH1090 (*pnc-1(uk212) IV*) was outcrossed 6 times, BLC298 (*sir-2*.*3(ok444) X; EX[zdls5 (pmec-4*::*GFP) I; mec-4(u231) X]; EX[SIR-2*.*3]; unc-122*::*GFP*), ZB164 *bzIs8 (p*_*mec-4*_*mec-4*::*GFP)* and BCL314 (*sir-2*.*3(ok444) X; p*_*mec-4*_*mec-4*::*GFP*), BLC321(*daf-16(mu86) I; EX[zdls5 (pmec-4*::*GFP) I; mec-4(u231) X]*), BLC324 (*daf-16(mu86) I; sir-2*.*3(ok444) X; EX[zdls5 (pmec-4*::*GFP) I; mec-4(u231) X]*), BLC325 (*pnc-1(uk212) IV*; *EX[zdls5 (pmec-4*::*GFP) I; mec-4(u231) X]*) BLC323 (*pnc-1(uk212) IV; sir-2*.*3(ok444) X; EX[zdls5 (pmec-4*::*GFP) I; mec-4(u231) X]*). Both VC199 (*sir-2*.*1(ok434) IV*) and RB654 (*sir-2*.*3(ok444) X*) were outcrossed 3 times. In *sir-2*.*1(ok434)*, *sir-2*.*3(ok444)* and *daf-16(mu86)* 768 bp (from nucleotide 501 to nucleotide 1268), 839 bp (from nucleotide 501 to nucleotide 1340) and 10650 bp (from nucleotide 12101 to nucleotide 22751) respectively are deleted.

### Molecular biology

For the sir-2.3 rescue construct, the 3700 bp sir-2.3 genomic DNA sequence was amplified from N2 genomic DNA using primers 5’-GGATCCCGGAACTTCATGGCAGTGCTCTTCAAGTA-3’ and 5’-GGTACCTGACATTTCTTTCAAAACATCCGAAATTCTGTAGTCTAACTTCATT-3’ that added BamHI and KpnI restriction sites to the 5’ and 3’ ends, respectively. *sir-2*.*3* genomic DNA was then cloned into pPD95.75 with the *sir-2*.*3* promoter. Germline transformation by microinjection was performed as described [72].

### Nematode synchronization

Gravid adults were collected in a 1.5 ml eppendorf tubes and treated with 200 μl of bleach plus 80 μl of 10M NaOH in 700 μl of water for ~ 7 min to release the eggs. After centrifugation for 3 min at 3000 rpm and removal of the supernatant, eggs were resuspended in 100 μl of sterile water, prior to inoculation onto seeded NGM plates.

### Fluorescent microscopy

Animals were mounted on thin agarose pads and immobilized by 20 mM Na-azide. Fluorescent micrographs were taken using a LEICA DMR2 fluorescent microscope equipped with 20X, 40X and 63X objective, a Spot RT slider camera (Diagnostic Instruments) equipped with Spot32 acquisition software, a LEICA green fluorescent protein (GFP) plus filter (460/480 nm excitation filter), and a LEICA rhodamine filter (535/550 nm excitation filter). For strict quantitative comparisons, images were acquired using the same exposure time (500 ms); images were analyzed and processed using ImageJ.

### Chemically induced ischemia and quantification of neuronal death

For chemical induced ischemia, synchronized 1 day old adult worms were exposed for 3h, 5h, 6h, and 8h at 22°C to the following solutions: 1) S Basal at pH 6.5 or 7.4 (for 100 ml of solution: 0.584 g NaCl, 0.1 g K2HPO4, 0.6 g KH2PO4, Acetic Acid 57 μl); 2) S basal pH 7.4 containing 20 mM Na-Azide; 3) S Basal at pH 6.5 plus 20 mM or 100 mM Na-Azide. 4) S Basal pH 6.8 plus 20 mM Na-Azide. After treatment, worms were allowed to recover on fresh NGM plates for 20 hours at 20°C and then stained with 2 mg/ml DiD [73] [36]. Animals were visualized under a 40X objective using rhodamine filters and DiD stained amphid sensory neurons were counted in each animal. The chemical ischemia protocol was modified from Scott and colleagues, 2002 [36] by using S Basal at pH 6.5 or 6.8 instead of 7.4 to mimic ischemic conditions in mammalian brain. Under these conditions, 0.91 +/- 0.029 proportion of wild type animals died, as compared to 0.245 +/- 0.064 at pH 7.4 (11 and 6 experiments respectively with at least 30 worms per experiment).

For quantification of neuronal death in *mec-4(d)* and *mec-10(d)* strains, GFP or mcherry expressing touch neurons were counted in synchronized L4-staged animals, and swollen PLM touch neurons were counted in synchronized L1-staged animals.

### *Caenorhabditis elegans* embryonic cell culture

Embryonic cell culture was performed as described previously [62]. Briefly, a large number of gravid adult worms were grown on enriched peptone agar plates (8P) with NA22 *Escherichia Coli*, collected in 50 ml tubes, washed 3 times with sterile H_2_O and centrifuged at 1200 rpm for 10 minutes. After removal of the supernatant, nematodes were transferred into 15 ml tubes and lysed with 5–6 ml of lysing solution (5 ml of fresh bleach, 1.25 ml of 10N NaOH and 18.5 ml of sterile H_2_O) for 5–10 min. The lysis was stopped by adding egg buffer (118 mM NaCl, 48 mM KCl, 2 mM CaCl_2_, 2 mM MgCl_2_, 25 mM Hepes, pH 7.3, 340 mOsm) to the tube. Lysed animals were then centrifuged for 10 minutes at 1,200 rpm. Eggs were separated from the animal carcasses using 2 ml of egg buffer plus 2 ml of 60% sucrose and centrifuged for 20 min at 1,200 rpm. Eggs floating at the top of the tube were collected in a new 15 ml tube with a P1000 pipetor, washed 3 times with fresh egg buffer and centrifuged for 10 min at 1,200 rpm. To dissociate the embryonic cells, eggs were incubated for 10–30 minutes with 1 ml of 2 mg/ml Chitinase (Streptomyces Griseus- Sigma Aldrich, C6137-25UN) dissolved in egg buffer pH 6.5. After enzymatic treatment, embryos were pelleted by centrifugation for 3 min at 2,500 rpm. The supernatant was removed and the eggs were resuspended in L-15 medium (Gibco, plus 10% Fetal Bovine Serum, 45 mOsm Sucrose, 1 U/ml Penicillin and 100 μg/ml Streptomycin). Cells were manually dissociated using a 10 ml syringe equipped with an 18 Gauge needle. The suspension containing cells and debris was subsequently filtered using a sterile 5 μM Millipore filter. Filtered cells were pelleted for 3 min at 2,500 rpm and resuspended in fresh L-15 medium. Cells were plated at ~230,000 cell/cm^2^ density in 24 wells plates on microscope slides (12 mm diameter) previously coated with 0.5 mg/ml peanut lectin (Sigma Aldrich, L0881-10MG). The media was replaced the day after and the cells were kept at 20°C for up to 7–9 days. For microscopy, cells were fixed for 15 min with 4% paraformaldehyde, washed three times with egg buffer, and mounted using Vectashield mounting medium.

### Quantitative real-time PCR

RNA was extracted from synchronized young adult *sir-2*.*3(ok444)* worms using TRIzol reagent (Invitrogen), following manufacture’s procedures. cDNA was synthesized using the High capacity RNA-to-cDNA Kit (Applied Biosystem). qRT-PCR was carried out using Taqman Universal Master Mix II (Applied Biosystem) and the average mRNA fold change of each target gene was calculated by comparing the CT (cycle threshold) of the target gene to that of the housekeeping gene *pmp-3*. All reactions had three technical replicates and each condition had three biological replicates. The Taqman probes span an exon junction: *sir-2*.*1* (3–4, 3–5), *sir-2*.*2* (5–6), *sir-2*.*3* (5–6), *sir-2*.*4* (2–3), *pmp-3* (3–4, 4–5). Relative quantification was with the ΔΔ*CT* method (2(−ΔΔ*CT*)), and *P* values were calculated by *t* test. The wild type was used as the calibrator to assess fold change in gene expression.

### Quantification of ROS

Adult *Caenorhabditis elegans* nematodes grown under standard conditions, starved for 48 hours (dietary deprivation, DD), or cultured on plates containing 5 mM 2-DG were subjected to chemical ischemia or incubated in S Basal for 2 hours. They were then incubated with 50μM H_2_-DCFDA (Sigma-Aldrich) and rocked for 1h at room temperature. The 100μM working solution in M9 was made from a 50mM stock solution in DMSO. Animals were then picked into M9 buffer for 10 minutes prior to being mounted for microscopy. Photographs were taken with a LEICA DMR2 fluorescent microscope using the same exposure time (800 ms). Fluorescence was then quantified using ImageJ.

### Statistics

We used Origin 6.1 or 9 for statistical analysis. Differences between groups were determined using t-Test (2 groups) or Anova with Bonferroni’s (multiple groups) as specified in each figure legend. P values are listed in the figure legends. Variance was similar between groups.

### Ethics statement

This study was conducted using *Caenorhabditis elegans*, which does not require approval from the Institutional Animal Care and Use Committee of the University of Miami. The research was performed following the ethical conduct rules of the University of Miami.
